# Do Native and Alien Species Differ in Their Ecological Strategies? A Test with Woody Plants in Tropical Rainforests on Réunion Island (Mascarene Archipelago, Indian Ocean)

**DOI:** 10.3390/plants12233990

**Published:** 2023-11-27

**Authors:** Lyse Heymans, Jean-Yves Meyer, Claudine Ah-Peng, Quentin Ethève, Olivier Flores, Christophe Lavergne, Bertrand Mallet, Hilde Parlevliet, Dominique Strasberg, Robin Pouteau

**Affiliations:** 1 AMAP, IRD, Pôle de Protection des Plantes, 97410 Saint-Pierre, Réunion, France; lyse.heymans@laposte.net (L.H.); quentinsciencethev@gmail.com (Q.E.); 2 University of Réunion, 97430 Le Tampon, Réunion, France; 3 Research Department, Government of French Polynesia, 98713 Papeete, Tahiti, French Polynesia; jean-yves.meyer@recherche.gov.pf; 4 PVBMT, University of Réunion, 97410 Saint-Pierre, Réunion, France; claudine.ahpeng@univ-reunion.fr (C.A.-P.); hildecparlevliet@gmail.com (H.P.); dominique.strasberg@univ-reunion.fr (D.S.); 5 OSU-R, University of Réunion, 97744 Saint-Denis, Réunion, France; 6 University Paul Sabatier Toulouse III, 31100 Toulouse, France; 7 Conservatoire Botanique National de Mascarin, 97436 Saint-Leu, Réunion, France; clavergne@cbnm.org (C.L.); bmallet@cbnm.org (B.M.); 8 Van Hall Larenstein University of Applied Sciences, 6882 CT Velp, The Netherlands

**Keywords:** biological invasion, elevational gradient, functional ecology, Grime’s CSR theory, Réunion, leaf trait

## Abstract

Understanding the mechanisms of biological invasions (e.g., competitive exclusion) is a key conservation challenge, especially on islands. Many mechanisms have been tested by comparing the characteristics of native and alien species, but few studies have considered ecological strategies. Here we aim at comparing the competitive ability, stress tolerance, and ruderalism (CSR) of native and alien trees in the tropical rainforests of Réunion Island. A total of sixteen 100 m^2^ plots (eight ‘near-trail’ and eight ‘off-trail’, at less disturbed sites) were established over a 2100 m elevational gradient. Three traits were measured in 1093 leaves from 237 trees: leaf area, leaf dry matter content and specific leaf area. They were converted into a CSR score assigned to each of the 80 surveyed tree species (70 native and 10 alien) using the ‘Stratefy’ ordination approach. C scores increased with basal area and S scores with elevation, but R scores were not higher along the trail, thus only partially validating Stratefy. Native and alien trees had similar CS strategies, thus challenging invasion hypotheses predicting a difference in ecological strategies and rather demonstrating the importance of environmental filtering. However, other differences falling outside the CSR theory may also explain the success of alien species on Réunion.

## 1. Introduction

Globalisation leads to the transport and spread of species beyond their area of origin, some of which naturalise, i.e., maintain self-sustaining populations in the wild without human intervention, and in some cases become invasive, i.e., pose threats to biodiversity [1]. The number of alien species continues to increase on most continents, with no sign of saturation in the last two centuries [2]. Thus, almost 4% of the world’s vascular flora (ca. 14,000 species) are now naturalised somewhere on the planet [3], and between 5 and 20% of alien species are considered invasive [4]. Invasive alien species are considered one of the best indicators of global biodiversity decline [5,6] and contributed to 60% of recent species extinctions in synergy with various other threats [7], particularly in island ecosystems [8]. Indeed, invasive alien plants have overwhelmed oceanic islands worldwide [9], to the point where the number of alien species now equals or exceeds the number of native and endemic species on many islands or archipelagos in the Indian and Pacific Oceans [10,11]. Therefore, there is an urgent need to better understand the mechanisms of biological invasions in order to better predict and mitigate their impacts.

To investigate the reasons for the success of alien species, the search for fundamental differences from native species is a widely used approach [12]. Habitats, i.e., the environment to which a species is adapted [13] and functional traits, i.e., measurable morphological, physiological or phenological characteristics that influence growth, reproduction, or survival [14] are probably among the most commonly compared attributes. Habitat comparisons between native and alien species have delivered little consensus. In the Mediterranean region, for example, invasive alien plants and native plants have been found to occupy the same variety of islands and habitats [15]. In New Zealand, the distribution of plants of different origin has been shown to be shaped by the same set of environmental variables, but native and alien plants separate out into different regions of the space defined by these variables [16]. In fact, the main drawback of the habitat approach is that it focuses on the outcome of the species interactions without considering the underlying mechanisms per se [17].

The functional approach is more mechanistic than the habitat-focused approach. Certain differences in functional traits, often associated with the ability to acquire resources, appear to increase the potential for invasiveness. For example, alien trees found in Argentina have been shown to have a higher specific leaf area (SLA) than native trees [18]. Other functional traits appear similar between alien and native species, such as the ability of many Australian plants to sequester carbon [19]. Overall, only a limited number of functional traits, such as those related to fecundity or resource use, appear to be systematically more important in invasive alien species [20]. However, values of isolated functional traits are not always adequate to assess in detail the different trade-offs in resource allocation.

A multidimensional trait-based approach promises a more integrative understanding of the differences between native and alien species [21]. Ecological strategies, i.e., combinations and trade-offs between different trait values, reflect the processes by which species acquire, invest in, and use resources to survive and increase their fitness and survival [22]. Species may thus have different combinations of functional traits to pursue the same ecological strategy [23]. The ecological strategy approach also has the potential to be easier to link to invasion hypotheses than approaches focused on separate traits.

The competition, stress tolerance, and ruderalism (CSR) theory for plants formulated by Grime [24] is among the most well-known theories related to ecological strategies. This theory suggests that vegetation dynamics and structure result from adaptive trade-offs among several correlated functional traits in response to competitive interactions, stress, and environmental disturbances [24,25]. Stress is defined as any constraint acting externally on vegetation that limits the production of dry matter on all or part of the plant [26]. Disturbance is described as a partial or complete destruction of plant biomass by herbivores, pathogens, humans, or climatic phenomena [26]. The evolutionary trade-offs between competition, resistance, and resilience to disturbance allow specific ecological strategies to be defined within an adaptive space consisting of three axes [27]. Competitors (C-strategists) survive in stable and productive habitats thanks to their ability to monopolise resources efficiently, especially through their spatial dynamics (large individuals and organs). Stress-tolerant plants (S-strategists) protect their metabolic performance in variable or resource-poor environments (often small individuals with dense and persistent tissues). They invest in their ability to conserve resources and withstand stress. Finally, ruderal plants (R-strategists) are pioneer species in disturbed areas (e.g., urban environments, wasteland, roadsides, agricultural fields) with rapid growth, a high reproductive rate, and long-distance dispersal [26,27]. Grime’s triangle provides a practical and quantitative approach to comparing plant functions and assessing how trade-offs between functional traits can facilitate the naturalisation or invasion of alien species [28,29].

Interestingly, some authors have proposed a standardised and generalisable method for positioning species within the CSR adaptive space based on ‘soft traits’, i.e., traits that are accessible and easily measurable in the field, such as leaf size and mass, and correlated with ‘hard traits’, i.e., traits related to basic physiology that are more difficult to access in the field, such as growth rate or leaf longevity [30,31,32,33]. In 2017, Pierce et al. developed a practical ordination tool calibrated at a global scale using thousands of vascular plant species (all life forms combined) called ‘StrateFy’ [34]. This tool estimates continuous CSR values based on the combination of three leaf traits: (1) leaf area (LA), which determines light interception capacity and is presumed to represent the size spectrum of the plant, (2) SLA, which reflects resource acquisition, and (3) leaf dry matter content (LDMC), which reflects resource conservation [34,35]. These three leaf traits are supposed to be representative of the variation in key functional traits of the whole plant [34,35,36]. So far, StrateFy has been mainly used at two levels of analysis: (1) either at the community level to assess the reliability of the method in predicting species assemblages, or (2) as a tool to compare the strategies of native and alien species. To our knowledge, however, no study has jointly considered the two levels.

Some authors have used StrateFy to identify how ecological strategies assemble within tree communities in contrasted environments [23,37,38,39]. In the subtropical forests of southern Brazil, temperature has been observed to influence the dominant ecological strategy of communities, being mainly C under warm conditions and S under cooler conditions [38]. In contrast, two other studies conducted at local scales have shown that tree communities in a resource-limited coastal ecosystem rather converge towards S/CS strategies [23,37].

More recently, three studies compared the ecological strategies of native and alien species using the StrateFy tool [34]. Dalle Fratte et al. compared, at a regional scale, in a temperate environment (Italy) and over a steep elevational gradient (from 10 to 4000 m a.s.l.), the differences in ecological strategies between different life forms [40]. Ecological strategies were found to be similar between native and alien non-woody plants. Alien trees were nevertheless significantly more competitive than native trees [40]. Furthermore, Guo et al. demonstrated that invasive alien species were globally more competitive than natives [28]. In another, more local study, Rojas-Sandoval et al. compared native and alien species in different forest types along a more modest elevation gradient on the tropical island of Puerto Rico [41]. Native and alien species showed converging ecological strategies in moist and wet forests but diverging strategies in dry forests, with aliens preferring a C strategy and natives an S strategy [41].

There are overall two opposing hypotheses on this subject. The ‘limiting similarity’ hypothesis proposes that the successful establishment of alien species would be unlikely if the native species of the recipient community hold similar functional traits and thereby similar resource acquisition strategies as the invader, resulting in increased competition for resources [42]. This means that aliens tend to differ phylogenetically or functionally from natives, which minimise competition [43]. This hypothesis is expected to result in native and alien woody plants diverging on one or more of the CSR axes. In contrast, the ‘environmental filtering’ hypothesis suggests that alien species need to be similar to native species occurring in the same habitat because a set of specific traits enables certain species, both native and alien, to establish and persist in a certain habitat [44]. This hypothesis is expected to result in converging CSR strategies.

Here, we combined the two approaches (analyses of ecological strategies at the community level along environmental gradients and among native and alien species) to answer the following questions: (1) Are the three soft leaf traits proposed by Pierce et al. robust in predicting the expected responses of ecological strategies along a triple environmental gradient of competition for space and resource availability, stress, and disturbance? Within a community and regardless of species origin, we would expect the C score to increase with forest stand basal area, the S score to vary with elevation (often associated with cold stress, insolation, or water deficit), and the R score to be higher under disturbed conditions; (2) Do native and alien species have diverging or converging ecological strategies? Given the local scale considered in our study, we expect the environmental filtering hypothesis to prevail over the limiting similarity hypothesis, which may rather act at a larger scale [43,45].

## 2. Materials and Methods

### 2.1. Study Site

This study was conducted on Réunion, the largest (2512 km^2^) and youngest island (2–3 million years old) of the Mascarene Archipelago (Indian Ocean), which also comprises the islands of Mauritius and Rodrigues. Islands are recognised as suitable sites to explain the ecological and evolutionary mechanisms underlying community assemblages [46]. Oceanic islands such as Réunion are also particularly sensitive to biological invasions, possibly as a result of the so-called ‘island syndrome’, which predicts a reduction in competitive and reproductive abilities in island species [47]. In addition, long-distance dispersal filters lead to oceanic islands being disproportionately poor (or rich) in certain taxonomic or functional groups compared to continents (‘disharmony’), which likely results in an unbalanced representation of certain traits in island species [48]. The study of ecological strategies offers an original perspective to understanding the interactions between native and alien island species, with the aim of predicting the success of alien species [49,50].

The climate of Réunion is tropical and characterised by two seasons: a warm and rainy austral summer in which the study was conducted (February–April 2023), followed by a relatively cool and dry winter [44]. Rainfall ranges from 500 mm/year on the leeward coast to >8000 mm/year on the windward coast (Appendix A). Réunion harbours a remarkable diversity of ecosystems zonated along an elevational gradient that extends from sea level to 3070 m a.s.l. on the slopes of the dormant volcano Piton des Neiges and to 2631 m on the lava flows of the active Piton de La Fournaise. The presence of strong environmental gradients over short distances theoretically makes the island a suitable study model for evaluating methods for estimating the ecological strategies of plants [34]. Although the island is one of the last places on Earth colonised by humans (350 years ago), its vegetation is profoundly influenced by anthropogenic impacts. Alien species, which include 474 plant taxa identified as invasive and 730 as potentially invasive, are among the main threats to native plants there [51].

### 2.2. Sampling Design

**Figure 1:**
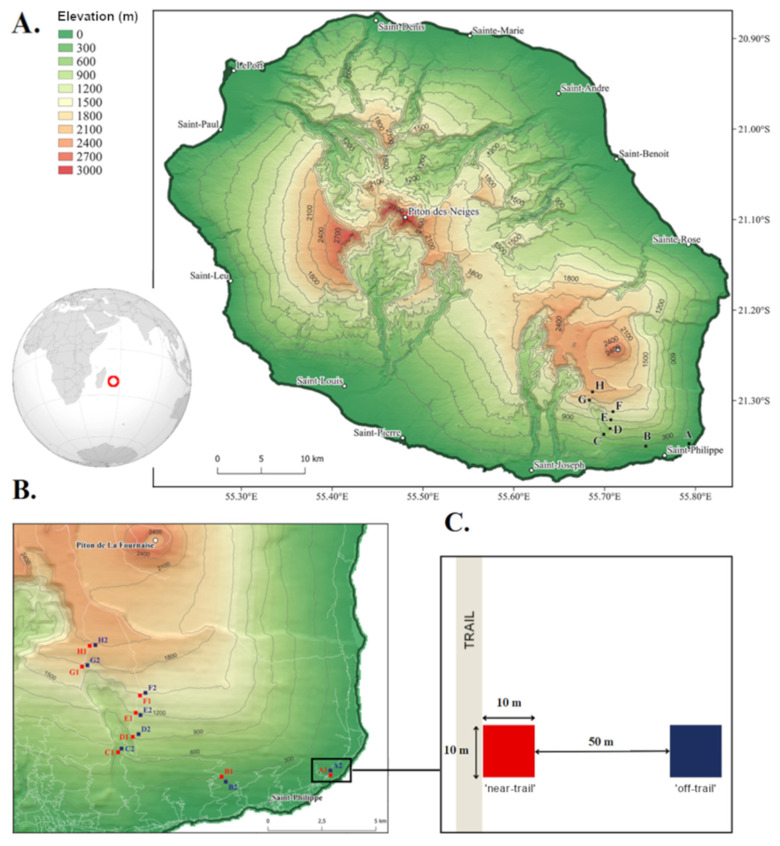
The study site. The red circle on the earth globe shows the location of Réunion within the Indian Ocean. (**A**) The topography of Réunion and the location of the plots (letters A to H) sampled in Saint-Philippe (on the southeastern side of the island). (**B**) A zoom in on the plots along an elevational gradient of 2100 m, from sea level to Piton de La Fournaise. The plots are established every 300 m, i.e., eight sampled elevations. (**C**) At each elevation, two 100 m^2^ plots located at the edge of the trail (‘near-trail’) and 50 m away (‘off-trail’) were inventoried.

A network of plots located from sea level (300 m from the coast) to the vicinity of Piton de La Fournaise at 2100 m was set up in the commune of Saint-Philippe, on the southeast coast of Réunion (Figure 1A). Precipitation throughout the study area is quite evenly distributed (with annual rainfall between 6000 and 7000 mm/year) [44]. However, the average annual temperature ranges from 12 to 26 °C, resulting in a pronounced zonation of the vegetation. Within the study area, four main forest types are found, which are anthropogenically modified to varying degrees. From sea level to 900 m, the vegetation is a typical lowland moist forest with large trees forming a relatively continuous canopy [52,53]. This habitat is the most affected by human activities (logging, agriculture, forest plantations, and urbanisation) and consists of a hybrid assemblage of alien (e.g., *Mangifera indica*, *Psidium cattleyanum*, and *Syzygium jambos*) and native plant species (e.g., *Labourdonnaisia calophylloides*, *Mimusops balata*). Then, a dense submontane wet forest dominated by *Casearia coriacea*, *Cordemoya integrifolia*, *Dombeya* spp., and *Molinaea alternifolia* is found between 900 m and 1300 m, followed by a montane cloud forest between 1300 m and 1800 m, with a high abundance of tree ferns (Cyatheaceae), palms (Arecaceae) and low, lying, and twisted trees such as *Monimia* spp. covered with epiphytes [52,53]. These mid- to high-elevation habitats are hardly affected by human activities. At the highest elevations, between 1800 m and 2100 m, the vegetation is subalpine and dominated by shrubs, mostly of the Ericaceae (*Erica reunionensis*) and Asteraceae families (*Hubertia* spp.).

Inventories were carried out at every 300 m of elevation along the gradient, resulting in eight elevational levels labelled from A (0 m) to H (2100 m, Figure 1B). The different trails running along the plot network (‘Jacques Payet’, GRR2, and the Mare-Longue forest track) were used as proxies of anthropogenic pressure whose effects on the importance of R strategies was tested. Thus, at each elevation, two 10 m × 10 m plots were established, one at the edge of the trail (‘near-trail’) and the other at least 50 m away (‘off-trail’) (Figure 1C). Trails have already been identified as corridors where alien species spread due to the opening up of the environment and the dispersal of seeds by hikers [54]. On Réunion, it has been shown that a large proportion of alien naturalised species were able to establish along trails in low-elevation rainforests [55]. This study is therefore based on a total of 16 plots of 100 m^2^.

### 2.3. Plot Structure and Composition

All woody individuals with a diameter at breast height (DBH) of 1 cm or more were considered in each 100 m^2^ plot. Palms, tree ferns, and pandanus meeting this criterion were also included. The basal area *G*, a measure of tree cover at breast height, was calculated for each plot as the sum of the basal area of all trees. The structural description of the vegetation was completed by measuring the maximum height (from the base of the trunk to the highest leaf of the highest branch) of each individual using a telescopic rod. To determine species composition, each tree was identified at the species level and given a unique number. Infraspecific taxa were grouped at the species level. The geographical origin of each species was based on the index of the vascular flora of Réunion [56].

### 2.4. Leaf Traits

All woody species (including trees, shrubs, monocot trees, and tree ferns, hereafter ‘trees’; Appendix B) were measured for leaf traits (LA, LDMC, SLA). Four adult individuals of each species were selected for leaf sampling to limit the effects of ontogeny. The four individuals were selected based on the elevational range occupied by each species (Appendix C). Two individuals were selected from the ‘near-trail’ plots, one at the lowest elevation at which the species occurs and the other at the highest elevation. Similarly, two individuals were sampled at the two extremes of the species elevational range among the ‘off-trail’ plots. This selection method makes it possible to obtain trait values representative of the species while accounting for the potential intraspecific variability of the leaves.

Five mature and healthy leaves (without symptoms of disease or signs of herbivory), exposed as much as possible to direct light (‘canopy’ leaves), were collected from each individual, i.e., a total of 20 leaves per species. The leaves of legally protected species were sampled by the two authors holding collecting permits (Christophe Lavergne and Bertand Mallet). Collected leaves were stored and transported as recommended by Pérez-Harguindeguy et al. [57]. Some leaf collections were abandoned because the trees to be sampled were either too high or had too few leaves (e.g., palms and tree ferns), which could have been lethal for them. In total, 17 species (21%) were affected by partially incomplete collection of individuals (seven species, 9%) and/or leaves (13 species, 16%).

LA was determined using LI-COR instruments consisting of a portable scanner (LI-3000C) embedded in a conveyor belt (LI-3050C). Each LA value was determined by the average of three repeated surface measurements of the same leaf to ensure accuracy. Due to their particular area, the fronds of tree ferns and palms as well as the small-scale-leaves of *Erica reunionensis* were analysed from photographs using the Gimp image editing program. Each leaf was weighed with a precision scale to determine the leaf water-saturated fresh mass (LFM), placed in an oven at 60 °C for at least 48 hours and re-weighed to determine the leaf dry mass (LDM) [57].

The LA, LFM, and LDM measured for each leaf were averaged at the individual level (average of the five leaves per individual) and then at the species level (average of the four individuals per species) to obtain a single value per species. These measurements were then used to calculate the SLA, defined as the ratio between LA and LDM, and the LDMC, defined as the ratio between LDM and LFM multiplied by 100. We then converted the three trait values (Appendix D) into three CSR scores for each species using the method of Pierce et al. [34]. These scores are calculated from the LA for the C score, the LDMC for the S score, and the SLA for the R score.

### 2.5. Data Analyses

First, we assessed the robustness of the three soft leaf traits for predicting local changes in ecological strategies based on environmental gradients specific to each axis of the CSR scheme. Competitiveness C was analysed as a function of the basal area of each plot, stress tolerance S as a function of elevation, and ruderalism R was compared between ‘near-trail’ and ‘off-trail’ plots. The similarity of the ‘near-trail’ and ‘off-trail’ communities was estimated at each elevation using the Sørensen index *S*_*sim*_ = 2*C*/(*A* + *B*), where *A* and *B* refer to the number of species from the ‘near-trail’ and ‘off-trail’ plots, and *C* is the number of species shared by the two plots [58]. Values of the Sørensen index of similarity range from 0 to 1, with 0 indicating no common species and 1 indicating that all species are common. Values above the threshold of 0.70 indicate that the species in the two communities are similar [58].

CSR values were analysed at the community level (i.e., by combining the CSR scores of all species in an assemblage) to determine whether the stand as a whole has dominant strategies related to the specific constraints of each environment. Alien species were therefore not separated from native species at this stage. Four levels of analysis were used to aggregate CSR values at the community level: (1) the C, S, and R scores of a community were estimated as the average of the scores for the species present in each plot. All species present were thus weighted equally, regardless of their abundance in the community; (2) CSR scores were weighted by the relative abundance of each species; (3) CSR scores were weighted by the relative stem density of each species (different from 2 when there are multi-stemmed individuals); (4) CSR scores were weighted according to the relative basal area of each species.

Logarithmic models were used to fit the C score as a function of basal area and third-order polynomial models were used to fit the S score as a function of elevation. The comparison of R scores between ‘near-trail’ and the ‘off-trail’ plots was carried out with a non-parametric Wilcoxon–Mann–Whitney test since the conditions of use of a parametric *t*-test were not fulfilled.

Second, each species was positioned in a CSR ternary plot using the R software package ‘ggtern’ v3.4.2 [59]. To examine whether each of the three CSR scores and functional traits (LA, LDMC, and SLA values) varied between native and alien species while controlling for phylogenetic distances, ANOVA were built using the *phylANOVA* function of the package ‘phytools’ v2.0-3 [60]. A phylogenetic tree was constructed for the 77 seed plant species (the two Mascarene-endemic tree ferns *Cyathea borbonica* and *C. excelsa*, and the Réunion-endemic *C. glauca* were excluded) using the ‘V.PhyloMaker’ package [61]. The default settings of the function *phylo.maker* (node = ‘build.nodes.1′ and scenarios = ‘S3′), which adds species missing from the backbone tree as polytomies in the middle of the branch of their genera, were used to build the tree. R scores and the LA and SLA values were log-transformed to approximate normality. *p*-values were obtained by phylogenetic simulation (i.e., simulating the trait on phylogeny using the Brownian motion model) with 10,000 runs and adjusted with a Bonferroni correction. *p*-values were found not to be sensitive to the unbalanced numbers of native and alien species (Appendix E).

## 3. Results

### 3.1. Plot Inventories

In a total area of 1600 m^2^, 80 different tree species with 1642 individuals and 2062 stems were inventoried. Of these species, 70 were native (including 30 island endemics and 29 archipelago endemics, of which seven are legally protected on Réunion) and 10 are alien (Appendix B). The latter were represented by 183 individuals (11%) and 227 stems (11%).

**Figure 2:**
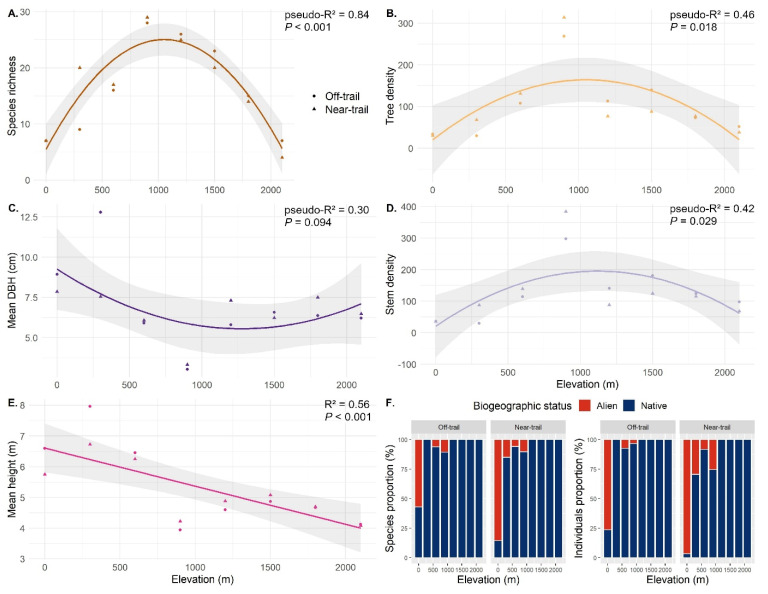
Change in structure and composition of the sixteen 10 m × 10 m plots established along a 2100 m elevational gradient in Saint-Philippe (Réunion). (**A**) Species richness (*n* = 80 species). (**B**) Tree density (*n* = 1642 trees). (**C**) Mean diameter at breast height (DBH) of all stems. (**D**) Stem density (*n* = 2062 stems). (**E**) Mean height of all individuals. (**F**) Proportion of native and alien species and individuals. Species richness, tree and stem densities, and mean DBH were fitted as a function of elevation using a second order polynomial model. Mean individual height was fitted with a simple linear model. The goodness of fit of each model is estimated using the explained variance pseudo-R^2^ and *p*-value. ‘Near-trail’ plots are represented by triangles and ‘off-trail’ plots by circles.

Plot species richness followed a hump-shaped pattern along the elevation gradient (pseudo-R^2^ = 0.84, *p* < 0.001, second order polynomial regression) with a maximum richness of 28–29 species/100 m^2^ reached at 900 m (Figure 2A). Tree density (pseudo-R^2^ = 0.46, *p* = 0.018, second order polynomial regression, Figure 2B) and stem density (pseudo-R^2^ = 0.42, *p* = 0.029, second order polynomial regression, Figure 2C) followed a similar hump-shaped trend as species richness and also peaked at 900 m with an average of 290 trees/100 m^2^ (Figure 2B) and 340 stems/100 m^2^ (Figure 2C), respectively. However, the average DBH followed the opposite tendency with a minimum of 3 cm at 900 m and a maximum of 6–8 cm at sea level and at the top of the gradient (pseudo-R^2^ = 0.30, *p* = 0.094, second order polynomial regression, Figure 2D). Finally, the mean height decreased linearly with elevation (R^2^ = 0.56, *p* < 0.001, linear regression, Figure 2E), reaching 6 m at 0–600 m and 4 m at 2100 m.

The proportion of alien species and individuals decreased with elevation (Figure 2F). At sea level, seven species were found in the ‘near-trail’ plot, of which six were alien, corresponding to 97% of the individuals (Figure 2F). This proportion was also very high in the ‘off-trail’ plot with four out of seven species being alien and 77% of individuals. Half of the alien species (five out of 10) occurred only in the sea-level plots with *Adenanthera pavonina*, accounting for half of the trees in the ‘near-trail’ plot, and *Mangifera indica*, accounting for one third of the trees in the ‘off-trail’ plot. Only a few individuals (between one and four) of the other three species (*Aleurites moluccanus*, *Artocarpus heterophyllus* and *Syzygium jambos*) were sampled, mostly in the ‘near-trail’ plot. At 300 m in the ‘near-trail’ plot (B1), among the 20 sampled species, only three (15%) were alien (Figure 2F). The proportion of alien individuals in B1 (29%) was mainly represented by *Psidium cattleyanum* while only one individual of *Litsea glutinosa* and another of *Schinus terebinthifolia* were inventoried (Figure 2F). No alien species were sampled in B2 (300 m, ‘off-trail’). In C1 and C2 (600 m), only one alien species (out of 17 species) was sampled: *Psidium cattleyanum*, which accounted for 8% of the individuals. In D1 (900 m, ‘near-trail’), a quarter (26%) of the individuals were alien (Figure 2F), mainly of the species *Psidium cattleyanum* and to a lesser extent *Rubus alceifolius* and *Ardisia crenata*. Plot D2 (900 m, ‘off-trail’) had only 3% of alien individuals. Above 900 m, no alien trees were observed in our plots. *Psidium cattleyanum* was the only alien species sampled from sea level to 900 m.

### 3.2. CSR Strategies of Trees on Réunion

**Figure 3:**
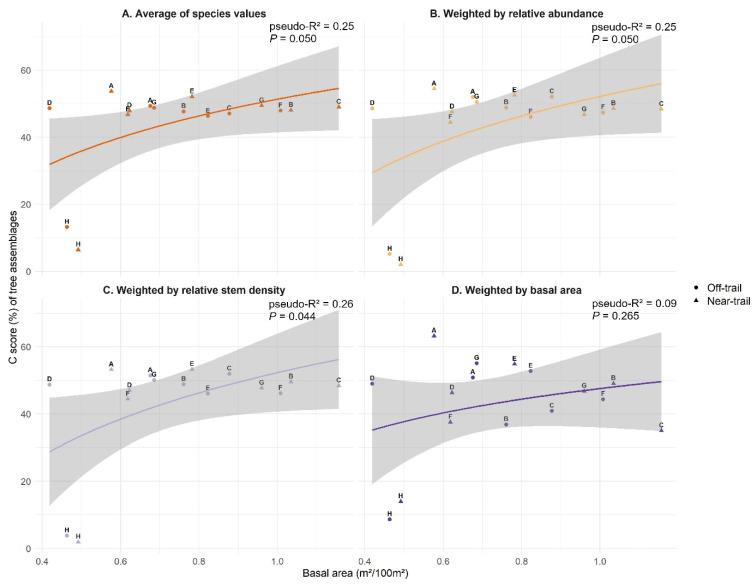
Competitiveness (C score) of tree assemblages sampled in Saint-Philippe (Réunion) as a function of stand basal area and for different community-level aggregation methods: (**A**) Average of species values (all species present have the same weight). (**B**) Average of species values weighted by their relative abundance. (**C**) Average of species values weighted by their relative stem density. (**D**) Average of species values weighted by their basal area. Logarithmic models were used to fit the data. The quality of the fit is estimated using the explained variance pseudo-R^2^ of and the *p*-value. ‘Near-trail’ plots are represented by triangles and ‘off-trail’ plots by circles. Plots have been identified with letters A to H.

The relationship between the community-level C score (average C score over all species in the assemblage) and the plot basal area was significant when the community-level C score was calculated by averaging the values of all species without weighting (Figure 3A), by weighting C scores by the relative abundance (Figure 3B) and by the relative stem density (Figure 3C). As for the best model (Figure 3C), the least competitive communities were H1 and H2 (2100 m), with average C scores of 2 and 4%, respectively, for a basal area below 0.50 m^2^/100 m^2^ (Figure 3C). The positive trend between C score and basal area seems largely due to these plots as there was no major variation in C score for higher values of basal area. In the other communities, average C scores varied from 44 to 53%. The most competitive communities were E1 (1200 m, ‘near-trail’) and A1 (sea level, ‘near-trail’) with average C scores of 53% for basal areas of 0.78 m^2^/100 m^2^ and 0.58 m^2^/100 m^2^, respectively. Plot C1 (600 m, ‘near-trail’) has the largest basal area of 1.16 m^2^/100 m^2^ and an average C score of 48 %. The other two communities with a basal area greater than 1 m^2^/100 m^2^ were plots B1 (300 m, ‘near-trail’) and F2 (1500 m, ‘off-trail’) whose average C scores were 50 and 46%, respectively (Figure 3C).

**Figure 4:**
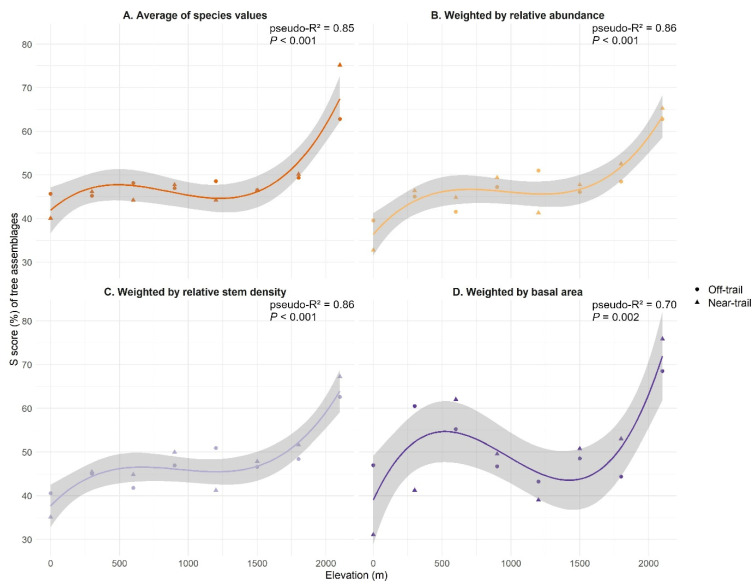
Stress tolerance (S score) of tree assemblages sampled in Saint-Philippe (Réunion) as a function of elevation and for different community-level aggregation methods: (**A**) Average of species values (all species present have the same weight). (**B**) Average of species values weighted by their relative abundance. (**C**) Average of species values weighted by their relative stem density. (**D**) Average of species values weighted by their basal area. Logarithmic models were used to fit the data. The quality of the fit is estimated using the explained variance pseudo-R^2^ of and the P-value. ‘Near-trail’ plots are represented by triangles and ‘off-trail’ plots by circles.

The community-level S score increased at increasing elevation with a plateau at mid-elevation (600–1500 m) at all levels of analysis (Figure 4). The lowest average S scores were found in the two plots at sea level A1 (34%) and A2 (40%). Subsequently, average S scores ranged from 41% to 53% between 300 m and 1800 m and reached their highest values at 2100 m with 63% in H2 (‘off-trail’) and 67% in H1 (‘near-trail’) (Figure 4B,C).

**Figure 5:**
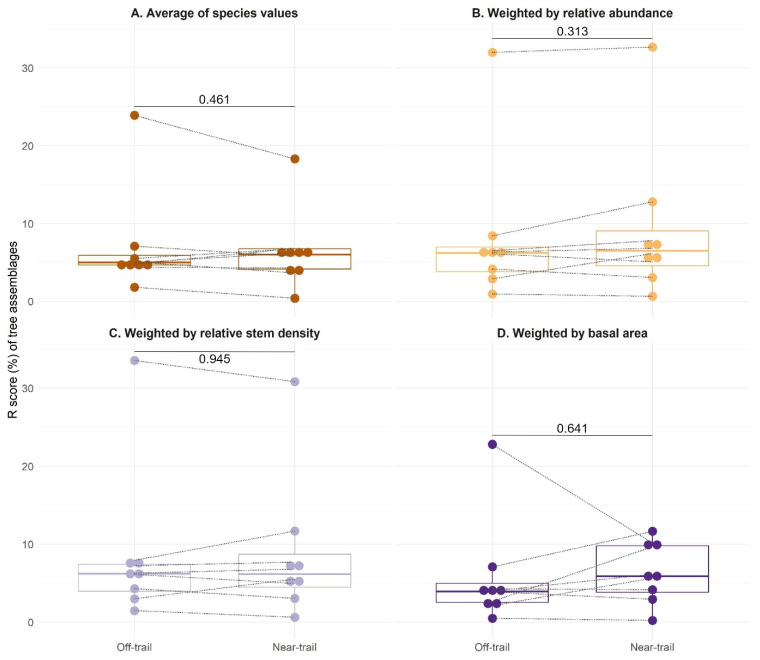
Ruderalism (R score) of tree assemblages sampled in Saint-Philippe (Réunion) according to their distance from the trail and for different methods of aggregating the values: (**A**) Average of species values (all species present have the same weight). (**B**) Average of species values weighted by their relative abundance. (**C**) Average of species values weighted by their relative stem density. (**D**) Average of species values weighted by their basal area. *p*-values of a non-parametric test (Wilcoxon–Mann–Whitney) comparing the means between ‘off-trail’ and ‘near-trail’ plots are indicated. The grey dotted lines represent the pairs of plots at each elevation.

Community-level R scores of ‘near-trail’ and ‘off-trail’ plots did not differ significantly regardless of the aggregation method used (Figure 5). The comparison with relative abundance weighting showed the largest differences between ‘near-trail’ and ‘off-trail’ plots (*p* = 0.31) (Figure 5B). According to this comparison, the median community-level R score for ‘near-trail’ and ‘off-trail’ plots was close to 7%. Values of Sørensen’s index of similarity between ‘near-trail’ and ‘off-trail’ plots ranged from *S*_*sim*_ = 0.28 at 600 m to 0.88 at 900 m with a mean along the elevational gradient of 0.68, indicating that the species in the two conditions largely overlap (Appendix F).

### 3.3. CSR Strategies of Native and Alien Tree Species on Réunion

**Figure 6:**
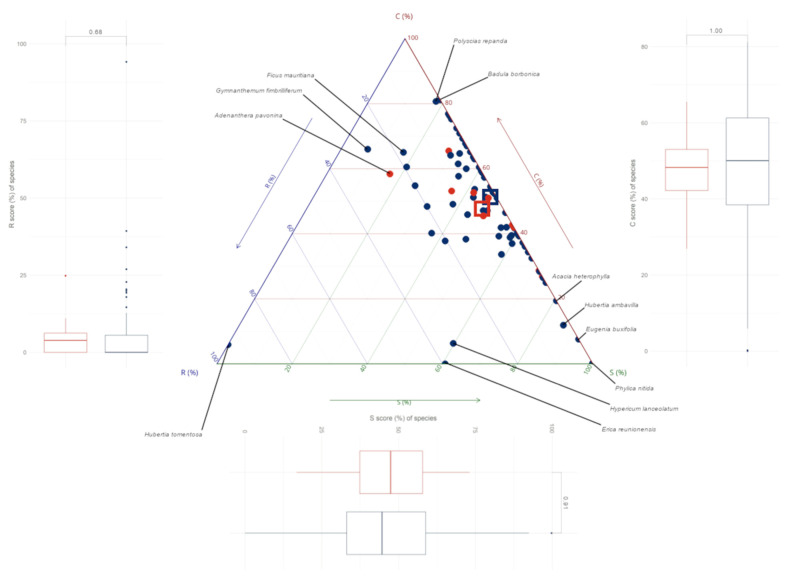
Comparison between the ecological strategies of native and alien tree species sampled in Saint-Philippe (Réunion). The three-dimensional diagram shows the CSR scores of native (blue circles, *n* = 70) and alien species (red circles, *n* = 10) and their respective median (blue and red squares, respectively). The C (red), S (green), and R (blue) axes represent the competitiveness, stress tolerance, and ruderalism of each species, respectively. The boxplots compare of the CSR scores obtained for native (blue, *n* = 67, i.e., without tree ferns) and alien species (red, *n* = 10). *p*-values of ANOVA accounting for the phylogeny in which the R scores were log-transformed are indicated.

About 70% of the 80 species had a CS strategy (Figure 6). Native and alien species had similar CSR scores, and the centroid of the two groups in the CSR space largely overlapped (Figure 6). Native and alien species also had similar LA, LDMC, and SLA values (Appendix G). Among the native species, most (about 70%) showed a compromise between the C and S strategies (scores in the range 30–70%) (Figure 6). However, some species lay outside this area of CSR triangles. On the C axis, two species were found to be particularly competitive: *Badula borbonica* and *Polyscias repanda* (C = 81% in both cases). On the S axis, *Phylica nitida* was the only species with a value of 100%, followed by *Eugenia buxifolia* (92%), *Hubertia ambavilla* (86%), and *Acacia heterophylla* (81%). At the opposite end of the axis, some species had S scores below 20%, namely *Hubertia tomentosa* (0%), *Gymnanthemum fimbrilliferum* (7%), and *Ficus mauritiana* and *Adenanthera pavonina* (17%). On the R axis, *Hubertia tomentosa* had by far the highest value (94%) followed by *Erica reunionensis*, *Hypericum lanceolatum*, and *Gymnanthemum fimbrilliferum* (R = 39, 34, and 27%, respectively).

Among the aliens, four species that occur only at sea level had a dominant C strategy (C > 51%): *Adenanthera pavonina*, *Aleurites moluccanus*, *Artocarpus heterophyllus*, and *Mangifera indica* (Table 1). At higher elevations, all alien species appeared to be better adapted to stress (S > C and R), with *Rubus alceifolius* being the only exception (S < C). The most commonly sampled alien species, *Psidium cattleyanum* (found from 0 to 900 m), had a mixed SC strategy (Table 1).

## 4. Discussion

The CSR ecological strategies derived from the Stratefy ordination method were aggregated for each woody plant community and associated with a specific environmental variable. Two of the anticipated correlations, i.e., an increase in competitive ability (C) with the basal area of the forest stand and an increase in stress tolerance (S) with elevation, were found to be significant, although disproportionately influenced by high-elevation communities (Figure 3 and Figure 4). However, ruderalism (R) was found not to be significantly higher in ‘near-trail’ plots than in ‘off-trail’ plots (Figure 5). The strategies of native and alien tree species were then compared and we showed that both groups share similar CSR trade-offs, irrespective of their origin, which results in a dominant CS strategy in our study site (Figure 6).

### 4.1. Assigned Ecological Strategies Were Only Partially Validated for Trees on Réunion

The use of basal area theoretically allows an indirect assessment of the intensity of competition for resources (e.g., space, light, nutrients) within a plot [62]. The increase in competitive ability as a function of basal area (Figure 3) therefore indicates that the C scores determined with the StrateFy method make sense overall. Community-level competitiveness was relatively high with the exception of the two highest-elevation plots (Figure 3). This can be explained by the fact that low- to mid-elevation tropical forests are usually relatively stable and productive, conditions that may favour competition between most woody species [27,34]. In contrast, there is a sharp transition zone between the montane cloud forest and the subalpine shrubland around 1800 m (pers. obs.), where plants might suddenly switch from a C to an S strategy.

Stress tolerance was studied as a function of elevation. Annual average temperature at sea level is around 24 °C over the study area and decreases linearly with elevation, reaching around 12 °C near Piton de La Fournaise, where the minimum temperature can be negative during the austral winter [44]. Temperature has already been identified as an environmental factor that strongly influences the ecological strategies of plants [38]. Our results show an increase in stress tolerance with elevation (Figure 4), suggesting that the S scores assigned by StrateFy are appropriate. This increase in stress tolerance is not linear, perhaps due to site effects (e.g., human land use, impact of cyclones or lava flows) or more positive species interactions (e.g., facilitation, symbioses) at species-rich mid-elevations (Figure 2A) [27,63]. Other oro-topographic stressors might also play a role in our gradient, such as exposure to sea spray at sea level, to wind in mountain ridges, or to high solar radiation at the highest elevations.

The expected difference in ruderalism between ‘near-trail’ and ‘off-trail’ plots was not confirmed for the tree stratum considered in our study. A possible explanation for this is that the trails of the study area are little disturbed, especially above 900 m [52,64], not very wide, which limits habitat fragmentation, and little used by hikers, which could explain the relatively low R scores that we found. In addition, sea-level plots contained many alien species of agricultural or forestry interest (*Mangifera indica*, *Aleurites moluccanus*, *Artocarpus heterophyllus*), whose distance from the trail may reflect a cultivation bias rather than a preference or not for ruderal conditions. Moreover, we noticed that the species with the highest R scores were native shrubs restricted to subalpine scrub (*Hubertia tomentosa*, *Erica reunionensis*, and *Hypericum lanceolatum*), which might reflect their adaptation to a natural regime of frequent disturbances (such as lava flows, fire, or cyclones) rather than to anthropogenic environments. Finally, the absence of alien trees above 900 m (Figure 2F) also reveals the good conservation of the study site, compared to other sites on Réunion, and may contribute to the difficulty of assessing the extent of R strategies.

### 4.2. Native and Alien Trees Use Similar Ecological Strategies on Réunion

Our comparison of ecological strategies between native and alien species revealed that the latter invade by occupying similar functional spaces to the former (Figure 6). This result tends to refute a number of invasion hypotheses that predict a difference in ecological strategies. The limiting similarity hypothesis, for instance, according to which a divergence between native and alien species on at least one of the CSR axes would have been expected, does not seem to apply to our study site. Indeed, niche differentiation and thus functional divergence appear to prevail at the regional scale more than at the local scale, where the role of environmental filtering and thus the convergence of functional traits seems more important [43,45].

Furthermore, alien species do not appear to be more competitive than native species (Figure 6A), contrary to what has sometimes been observed in temperate climates [40]. Therefore, our findings tend not to support the ‘global competition’ or ‘evolution of increased competitive ability’ hypotheses. These assumptions have often been used to explain the high degree of invasion of oceanic islands [20], as island species are often considered to be less competitive than continental species due to their isolated evolutionary history [65]. However, as argued by Barton and Fortunel, there does not appear to be a prevailing trend demonstrating the competitive inferiority of native island species compared to alien continental species [49]. The measure of competitiveness proposed by Pierce et al. is based on a single trait, LA, which correlates with canopy height and other reproductive traits (namely, seed mass, and volume). Yet, a meta-analysis by van Kleunen et al. showed that invasive alien plants do not have higher values for LA allocation, unlike many other performance-related traits [66]. The raw LA values that we measured on Réunion’s trees (from which C scores were derived) were also similar between native and alien species (Appendix F).

Other potentially beneficial traits for competition that were not considered in the StrateFy approach could explain the success of some alien species [67]. Traits related to reproduction and dispersal appear to play a key role in the success of the alien species studied. For example, the high reproductive capacity (associated with an early reproductive age, a large number of seeds produced and/or a continuous production of flowers and fruits) is known in many species such as *Schinus terebinthifolia*, *Adenanthera pavonina*, and *Ardisia crenata* [68,69]. The massive production of attractive fleshy fruits also means that frugivores (especially birds) prefer to disperse them [70], as this is clearly the case with *Psidium cattleyanum*, which reduces at the same time the dispersal of native species [71].

The majority of species, regardless of their origin, converged towards a CS strategy in our study site (Figure 6). This may support the prediction of abiotic environmental filtering of species leading to functional similarity between native and alien species [72]. The study by Rojas-Sandoval et al. reached the same conclusion that native and alien species share the CS functional space in similar tropical ecosystems (mesic to wet forests) [41]. Moreover, global data from Pierce et al. confirm the predominance of an intermediate CS strategy for trees in tropical forests [34].

Nevertheless, the convergence of ecological strategies between native and alien species may actually hide marked differences that the StrateFy approach is not able to capture. The use of three leaf morphological traits is useful and attractive for embracing and comparing a wide range of plants along environmental gradients [34]. However, considering different types of independent (i.e., uncorrelated) functional traits that relate to different organs often proves important for a more realistic assessment of strategies [37,73]. For instance, clonal propagation is common in the alien species that we inventoried (e.g., *Psidium cattleyanum*, *Rubus alceifolius*, *Schinus terebinthifolia*, *Litsea glutinosa*, *Ardisia crenata*) and likely contributes to their success. As an example, alien clonal plants were found to be more competitive in terms of nitrogen monopolisation than native clonal plants under controlled conditions in China [74]. Moreover, allelopathic characteristics are exhibited by several alien species sampled in our study site (e.g., *Psidium cattleyanum*, *Schinus terebinthifolia*), which potentially limits native species recruitment [75,76] and might explain the success of some of these alien species [77]. Interestingly, the most successful alien tree in our study site (*Psidium cattleyanum*) exhibits both clonal recruitment and allelopathic leaves [78]. Mutualisms such as associations with soil microorganisms [79] could also facilitate the establishment of some of the alien plants studied, such as the formation of mycorrhizae, which facilitates resource acquisition in *Ardisia crenata* [80].

Our comparison of native and alien tree species on Réunion showed no difference in stress tolerance (Figure 6B). Although the study area offers many advantages for this type of study (e.g., a forest continuum along a strong elevation gradient on a small spatial scale with homogeneous rainfall), our 10 alien tree species certainly did not provide a complete picture of the diversity in ecological strategies developed by the alien flora on Réunion. Indeed, the study area is dominated by extremely wet forests, which are potentially less favourable for many of the worst invasive alien plants [64]. Repeating this study on the same elevation gradient, but this time on the leeward coast (north-west), would make it possible to broaden the range of habitats (including semi-dry to mesic forests) and human-induced disturbances considered. This would also allow us to compare a larger number of alien species and measure the general scope of our results. Finally, only woody species (including trees, shrubs, monocot trees, and tree ferns) were considered in this study. The inclusion of the herbaceous layer, which also includes many problematic alien species, such as those belonging to the Poaceae, Melastomataceae, or Begoniaceae families [64,81], would allow for further analyses on differences in ecological strategies (especially ruderalism) between life forms [29].

## 5. Conclusions

The study of plant ecological strategies provides a multidimensional approach to functional traits that is more integrative in terms of understanding the mechanisms of biological invasions. Here we used an ordination approach to assign ecological strategies based on three soft leaf traits of native and alien woody species. Competitive ability and stress tolerance were positively correlated with the intensity of competition (basal area) and cold stress (elevation), respectively. However, ruderalism was not associated with the variable that we selected to estimate the level of anthropogenic disturbance (distance from trails). We then showed that the CSR adaptive space is similarly occupied by native and alien species over our study area, which does not support the hypothesis that alien species are more competitive or benefit from vacant or under-exploited ecological niches, and rather suggests a key role of environmental filtering. Nevertheless, other traits that do not fall within the theoretical framework of Grime’s CSR theory could explain the success of alien species such as clonal reproduction or allelopathy. Further studies in other ecosystems and on non-woody plants are needed to reinforce our finding that native and alien plants share common ecological strategies.

## Figures and Tables

**Table 1:** Elevational distribution and relative abundance (‘near-trail’ and ‘off-trail’ plots taken together) of the 10 sampled alien tree species with their associated ecological strategies.

Alien Species	Elevation (m)	Relative Abundance (%)	C:S:R (%)	Ecological Strategy
*Adenanthera pavonina*	0	38	58:17:25	C/CSR
*Aleurites moluccanus*	0	5	65:29:06	C/CS
*Artocarpus heterophyllus*	0	2	53:36:11	CS/CSR
*Mangifera indica*	0	23	51:47:02	CS
*Syzygium jambos*	0	3	42:58:00	CS
*Litsea glutinosa*	300	1	27:73:00	S/CS
*Schinus terebinthifolia*	300	1	45:48:06	CS
*Ardisia crenata*	900	1	29:71:00	S/CS
*Rubus alceifolius*	900	5	53:42:05	CS
*Psidium cattleyanum*	0; 300; 600; 900	16; 18; 8; 10	43:57:00	CS

**Figure A1:**
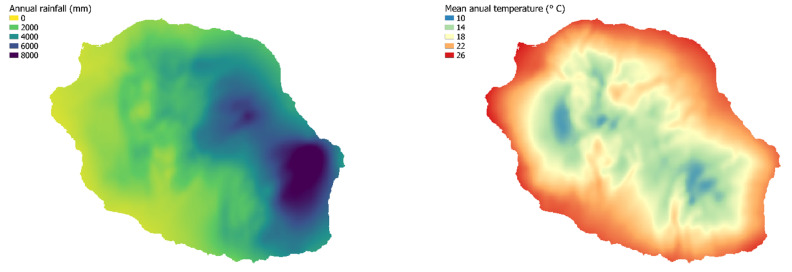
Distribution of annual rainfall (mm) and mean annual temperature (°C) over Réunion Island (adapted from Réchou et al., 2019 [44]).

**Figure A2:**
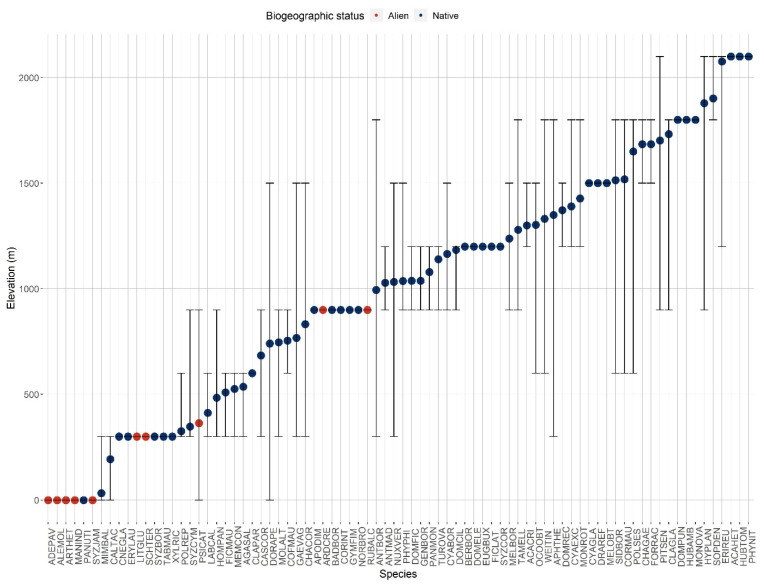
Elevational range at which each species occurs within our network of plots set up in Saint-Philippe (Réunion) (*n* = 80). Vertical bars indicate minimum and maximum elevations. Species are ranked by increasing mean elevation (blue dots for native woody plants and red dots for aliens). Species ID (the first three letters of the genus followed by the first three letters of the species in Latin) are given in Appendix B.

**Figure A3:**
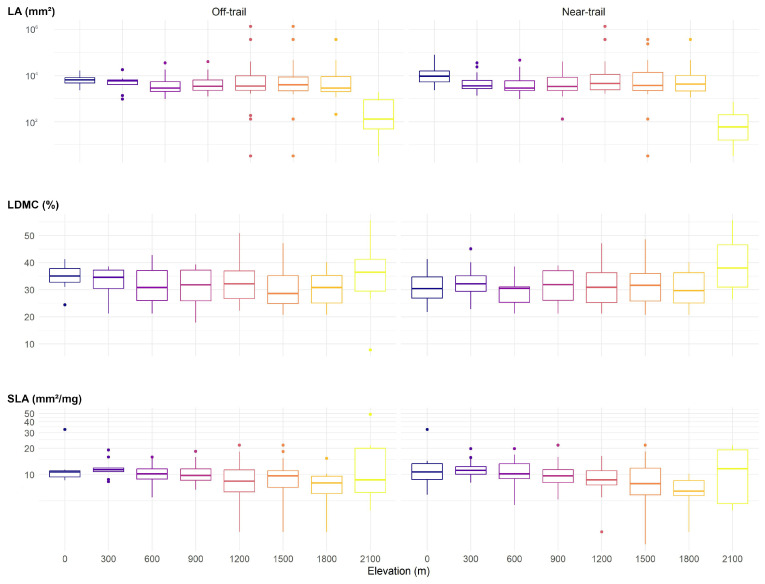
Boxplots showing the distribution of the three leaf functional traits that we measured in the field as a function of elevation and distance from the trail (‘off-trail’ or ‘near-trail’).

**Figure A4:**
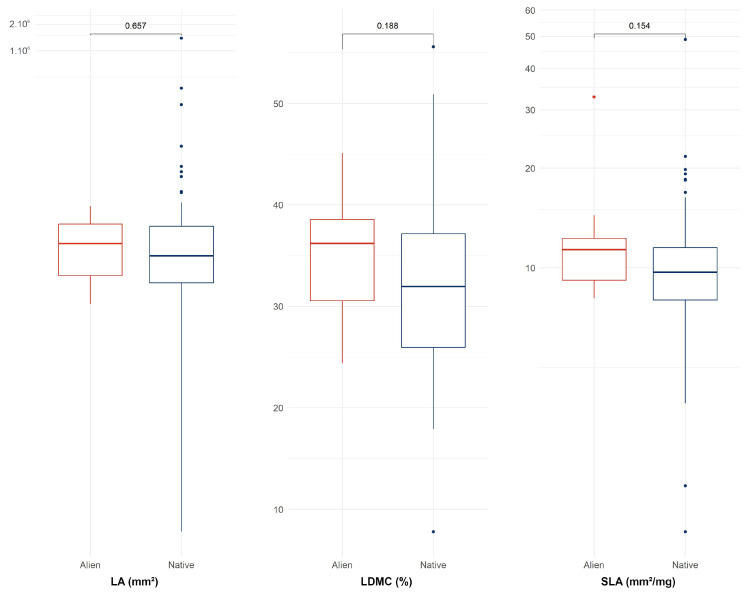
Comparison of three leaf functional traits between native (blue, *n* = 67, i.e., without tree ferns) and alien woody species (red, *n* = 10). *p*-values of ANOVA accounting for phylogeny in which the LA and SLA values were log-transformed are indicated.

## Data Availability

Data are contained within the article.

## Appendix B

**Table A1:** List of the 80 woody species sampled in Saint-Philippe (Réunion) for this study. Local IUCN status: DD = data deficient, LC = least concern, NT = near threatened, VU = vulnerable, EN = endangered. Biogeographic status (taken from Boullet 2020 [56]): R = Réunion, M = Mascarenes, IO = Indian Ocean. Habit: T = small and large trees; S = shrubs and subshrubs; F = tree ferns; P = palms, V = vines.

Species	Family	IUCN Status	Protected	Biogeographic Status	Habit	Species ID
*Mangifera indica*	Anacardiaceae		-	Alien	T	MANIND
*Schinus terebinthifolia*	Anacardiaceae		-	Alien	T	SCHTER
*Xylopia richardii*	Annonaceae	LC	X	Endemic (M)	T	XYLRIC
*Aphloia theiformis*	Aphloiaceae	LC	-	Native	T	APHTHE
*Tabernaemontana mauritiana*	Apocynaceae	NT	-	Endemic (M)	S	TABMAU
*Polyscias* cf. *sessiliflora*	Araliaceae	EN	X	Endemic (R)	S	POLSES
*Polyscias repanda*	Araliaceae	LC	-	Endemic (R)	T	POLREP
*Acanthophoenix crinita*	Arecaceae	VU	-	Endemic (R)	P	ACACRI
*Cordyline mauritiana*	Asparagaceae	LC	-	Endemic (M)	S	CORMAU
*Dracaena reflexa*	Asparagaceae	LC	-	Endemic (IO)	T	DRAREF
*Gymnanthemum fimbrilliferum*	Asteraceae	LC	-	Endemic (R)	S	GYMFIM
*Hubertia ambavilla*	Asteraceae	LC	-	Endemic (M)	S	HUBAMB
*Hubertia tomentosa*	Asteraceae	LC	-	Endemic (R)	S	HUBTOM
*Memecylon confusum*	Bois de balai	LC	-	Endemic (R)	S	MEMCON
*Calophyllum tacamahaca*	Calophyllaceae	LC	-	Endemic (M)	T	CALTAC
*Cnestis glabra*	Connaraceae	LC	-	Endemic (IO)	V	CNEGLA
*Weinmannia tinctoria*	Cunoniaceae	LC	-	Endemic (M)	T	WEITIN
*Cyathea borbonica*	Cyatheaceae	LC	-	Endemic (M)	F	CYABOR
*Cyathea excelsa*	Cyatheaceae	LC	-	Endemic (M)	F	CYAEXC
*Cyathea glauca*	Cyatheaceae	LC	-	Endemic (R)	F	CYAGLA
*Agarista salicifolia*	Ericaceae	LC	-	Native	T	AGASAL
*Erica reunionensis*	Ericaceae	LC	-	Endemic (R)	S	ERIREU
*Erythroxylum laurifolium*	Erythroxylaceae	LC	-	Endemic (M)	S	ERYLAU
*Forgesia racemosa*	Escalloniaceae	LC	-	Endemic (R)	S	FORRAC
*Mangifera indica*	Anacardiaceae		-	Alien	T	MANIND
*Aleurites moluccanus*	Euphorbiaceae		-	Alien	T	ALEMOL
*Claoxylon glandulosum*	Euphorbiaceae	LC	-	Endemic (R)	S	CLAGLA
*Claoxylon parviflorum*	Euphorbiaceae	LC	-	Endemic (M)	S	CLAPAR
*Cordemoya integrifolia*	Euphorbiaceae	LC	-	Endemic (M)	T	CORINT
*Acacia heterophylla*	Fabaceae	LC	-	Endemic (R)	T	ACAHET
*Adenanthera pavonina* var. *pavonina*	Fabaceae		-	Alien	T	ADEPAV
*Sophora denudata*	Fabaceae	EN	X	Endemic (R)	S	SOPDEN
*Hypericum lanceolatum*	Hypericaceae	LC	-	Endemic (IO)	S	HYPLAN
*Litsea glutinosa*	Lauraceae		-	Alien	T	LITGLU
*Ocotea obtusata*	Lauraceae	LC	-	Endemic (M)	T	OCOOBT
*Geniostoma borbonicum*	Loganiaceae	LC	-	Endemic (M)	S	GENBOR
*Dombeya ciliata*	Malvaceae	LC	-	Endemic (R)	T	DOMCIL
*Dombeya elegans*	Malvaceae	LC	X	Endemic (R)	S	DOMELE
*Dombeya ficulnea*	Malvaceae	LC	-	Endemic (R)	T	DOMFIC
*Dombeya punctata*	Malvaceae	LC	-	Endemic (R)	T	DOMPUN
*Dombeya reclinata*	Malvaceae	LC	-	Endemic (R)	T	DOMREC
*Turraea ovata*	Meliaceae	VU	-	Endemic (M)	S	TUROVA
*Apodytes dimidiata*	Metteniusaceae	VU	-	Native	T	APODIM
*Monimia ovalifolia*	Monimiaceae	LC	-	Endemic (M)	S	MONOVA
*Monimia rotundifolia*	Monimiaceae	LC	-	Endemic (R)	S	MONROT
*Tambourissa elliptica*	Monimiaceae	LC	-	Endemic (R)	T	TAMELL
*Artocarpus heterophyllus*	Moraceae		-	Alien	T	ARTHET
*Ficus lateriflora*	Moraceae	LC	-	Endemic (M)	T	FICLAT
*Ficus mauritiana*	Moraceae	LC	-	Endemic (M)	T	FICMAU
*Eugenia buxifolia*	Myrtaceae	LC	-	Endemic (R)	S	EUGBUX
*Psidium cattleyanum*	Myrtaceae		-	Alien	S	PSICAT
*Syzygium borbonicum*	Myrtaceae	EN	X	Endemic (R)	T	SYZBOR
*Syzygium cordemoyi*	Myrtaceae	LC	-	Endemic (R)	S	SYZCOR
*Syzygium cymosum*	Myrtaceae	LC	-	Endemic (M)	S	SYZCYM
*Syzygium jambos*	Myrtaceae		-	Alien	T	SYZJAM
*Noronhia broomeana*	Oleaceae	LC	-	Endemic (M)	T	NORBRO
*Pandanus montanus*	Pandanaceae	LC	-	Endemic (R)	S	PANMON
*Pandanus utilis*	Pandanaceae	LC	-	Native	T	PANUTI
*Antidesma madagascariense*	Phyllanthaceae	LC	-	Endemic (IO)	S	ANTMAD
*Phyllanthus phillyreifolius*	Phyllanthaceae	LC	-	Endemic (M)	S	PHYPHI
*Pittosporum senacia*	Pittosporaceae	LC	-	Endemic (IO)	S	PITSEN
*Ardisia crenata*	Primulaceae		-	Alien	S	ARDCRE
*Badula borbonica*	Primulaceae	LC	X	Endemic (R)	S	BADBOR
*Phylica nitida*	Rhamnaceae	LC	-	Endemic (M)	S	PHYNIT
*Rubus alceifolius*	Rosaceae		-	Alien	V	RUBALC
*Antirhea borbonica*	Rubiaceae	LC	-	Endemic (IO)	T	ANTBOR
*Bertiera borbonica*	Rubiaceae	DD	X	Endemic (R)	S	BERBOR
*Chassalia corallioides*	Rubiaceae	LC	-	Endemic (R)	S	CHACOR
*Chassalia gaertneroides*	Rubiaceae	LC	-	Endemic (R)	S	CHAGAE
*Coffea mauritiana*	Rubiaceae	LC	-	Endemic (M)	S	COFMAU
*Gaertnera vaginata*	Rubiaceae	LC	-	Endemic (R)	S	GAEVAG
*Melicope borbonica*	Rutaceae	LC	-	Endemic (R)	S	MELBOR
*Melicope obtusifolia*	Rutaceae	LC	-	Endemic (M)	T	MELOBT
*Casearia coriacea*	Salicaceae	LC	-	Endemic (M)	S	CASCOR
*Homalium paniculatum*	Salicaceae	LC	-	Endemic (M)	T	HOMPAN
*Doratoxylon apetalum*	Sapindaceae	LC	-	Endemic (IO)	T	DORAPE
*Molinaea alternifolia*	Sapindaceae	LC	-	Endemic (M)	T	MOLALT
*Labourdonnaisia calophylloides*	Sapotaceae	LC	-	Endemic (M)	T	LABCAL
*Mimusops balata*	Sapotaceae	LC	-	Endemic (M)	T	MIMBAL
*Sideroxylon borbonicum*	Sapotaceae	LC	-	Endemic (R)	T	SIDBOR

## Appendix E. Procedure Used to Test the Sensitivity of Our Comparisons between Native and Alien Species to Their Unbalanced Numbers

As we collected much more native than alien species, we simulated balanced datasets by randomly selecting the same number of native species as alien species to be inputted in the phylogenetic ANOVA, and repeated this procedure 999 times. Based on these simulations, we then computed the probability of arriving at no significant difference between native and alien species, i.e., a *p*-value higher than 0.05. In all cases (namely, comparisons of CSR scores, LA, LDMC, and SLA), this probability was 1. This means that native and alien species have similar strategies and trait values whatever the randomly selected native species.

## Appendix F

**Table A2:** Values of the Sørensen’s index of similarity *S* between ‘near-trail’ and ‘off-trail’ communities at each elevation. *A* and *B* refer to the number of species from the ‘near-trail’ and ‘off-trail’ plots, respectively, and *C* is the number of species shared by the two plots. *S* is expressed by *S* = 2*C*/(*A* + *B*).

Elevation (m)	*A*	*B*	*C*	*S*_*sim*_
0	7	7	4	0.57
300	20	29	4	0.28
600	17	16	12	0.73
900	29	28	25	0.88
1200	25	26	18	0.71
1500	20	23	15	0.70
1800	14	15	12	0.83
2100	4	7	4	0.73
